# Dopamine Modulates Acetylcholine Release via Octopamine and CREB Signaling in *Caenorhabditis elegans*

**DOI:** 10.1371/journal.pone.0072578

**Published:** 2013-08-16

**Authors:** Satoshi Suo, Shoichi Ishiura

**Affiliations:** Department of Life Sciences, Graduate School of Arts & Sciences, University of Tokyo, Tokyo, Japan; Brown University/Harvard, United States of America

## Abstract

Animals change their behavior and metabolism in response to external stimuli. cAMP response element binding protein (CREB) is a signal-activated transcription factor that enables the coupling of extracellular signals and gene expression to induce adaptive changes. Biogenic amine neurotransmitters regulate CREB and such regulation is important for long-term changes in various nervous system functions, including learning and drug addiction. In *Caenorhabditis elegans*, the amine neurotransmitter octopamine activates a CREB homolog, CRH-1, in cholinergic SIA neurons, whereas dopamine suppresses CREB activation by inhibiting octopamine signaling in response to food stimuli. However, the physiological role of this activation is unknown. In this study, the effect of dopamine, octopamine, and CREB on acetylcholine signaling was analyzed using the acetylcholinesterase inhibitor aldicarb. Mutants with decreased dopamine signaling exhibited reduced acetylcholine signaling, and octopamine and CREB functioned downstream of dopamine in this regulation. This study demonstrates that the regulation of CREB by amine neurotransmitters modulates acetylcholine release from the neurons of *C. elegans*.

## Introduction

The transcription factor cAMP response element binding protein (CREB) binds cAMP response element (CRE) and, upon activation by phosphorylation, induces the expression of downstream genes [1]. Biogenic amine neurotransmitters, including dopamine, serotonin, and norepinephrine, have been shown to regulate CREB activation through G protein-coupled receptors and G protein-mediated signaling [2], [3]. The amine-mediated regulation of CREB plays important roles in many biological processes, including learning and drug addiction. CREB has been shown to regulate a large number of neuronally enriched genes, including genes that function in neurotransmitter or growth factor signaling, and genes encoding transcription or signal transduction factors [4]. These genes have important roles in the regulation of neuronal development, plasticity, and protection, and, although the precise mechanisms are not entirely known, CREB induces long-term changes in the condition of the neurons in which it is activated.

In the model animal *Caenorhabditis elegans*, the *crh-1* gene, which encodes CREB [5], is required for long-term learning in various experimental paradigms, as seen in other animals [6]–[9]. *crh-1* also controls the expression of *tph-1*, which is required for serotonin synthesis [10], and plays a role in the regulation of aging [11]. We previously showed that CREB was regulated by biogenic amines in *C. elegans* using the *cre::gfp* reporter [12], [13], in which a CRE sequence is fused to GFP sequence. This reporter allows for the detection of CRE-mediated gene expression through GFP fluorescence in living animals [5]. Using this reporter system, we found that CREB was activated in all four cholinergic SIA neurons of *C. elegans* in the absence of food [12]. This activation is mediated by an amine neurotransmitter called octopamine, which is considered to be the biological equivalent of norepinephrine in invertebrates [14]. The octopamine receptor SER-3 and Gq alpha subunit EGL-30 function in SIA neurons to induce activation of the CREB homolog CRH-1. We subsequently found that dopamine signaling, which is believed to be activated in the presence of food in *C. elegans*, suppressed CREB activation in SIA neurons by inhibiting octopamine signaling [13]. Dopamine works through the dopamine receptors DOP-2 and DOP-3 and Gi/o alpha subunit GOA-1 to suppress octopamine release from octopaminergic neurons as well as octopamine-induced signaling in SIA neurons. However, the physiological role of CREB activation in SIA neurons is unknown.

Since SIA neurons are known to be cholinergic [15], it is possible that CREB activation in these neurons plays a role in the regulation of acetylcholine signaling. In this study, we examined acetylcholine signaling by monitoring aldicarb sensitivity and found that the regulation of CREB activation by biogenic amines in SIA neurons modifies acetylcholine signaling.

## Materials and Methods

### Strains

The culturing and genetic manipulation of *C. elegans* were performed as described [16]. The alleles used in this study were; *ser-3(ad1774) I* [12] (a gift from Drs. T. Niacaris and L. Avery, University of Texas Southwestern Medical Center, Dallas, TX), *cat-2(e1112) II* [17], *cat-2(tm2261) II* [18] (a gift from the National BioResource Project [NBRP], Ministry of Education, Culture, Sports, Science and Technology [MEXT], Tokyo, Japan), *crh-1(tz2) III* [5], *dop-2(vs105) V* [19], *tbh-1(ok1196) X* [12], and *dop-3(vs106) X* [19]. Double mutants were made using standard crossing techniques. The genotypes were confirmed by PCR for the deletion mutants and by PCR-RFLP for *cat-2(e1112)*.

### Aldicarb and levamisole assays

The measurement of aldicarb and levamisole sensitivity was conducted as described [20] with some modifications. Aldicarb (AccuStandard Inc., New Haven, CT) and levamisole (Sigma-Aldrich, St. Louis, MO) were dissolved in DMSO and added to molten NGM agarose (standard NGM agar except that the agar was replaced by agarose) at a final concentration of 1 and 0.2 mM, respectively. Aliquots of 2 ml of each were transferred to 35-mm Petri dishes and allowed to solidify. An overnight culture of the bacterial strain OP50 in LB medium was diluted 20 times with water. A total volume of 20 µl of the diluted bacteria was then spread over the plates, except when the animals were tested in the absence of food. The plates were allowed to dry without lids for at least 1 h and stored at 20°C overnight to grow the bacteria. To prepare the animals used in the assays, an adult animal was placed on an NGM plate seeded with OP50. The plate was incubated at 20°C for 4 days to allow most of the F1s to become adults. Approximately 25 adult animals on the culture plates were transferred with a platinum wire to an aldicarb or levamisole assay plate. The animals were examined every 15 min and scored as paralyzed if they did not move after being prodded with a platinum wire. The experimenter was blinded to the genotypes of the tested animals. Each assay was done in duplicate and repeated at least four times.

### Transgenic strains

The primers used for fusion gene construction were as follows (from 5′ to 3′):

A, ggatccaccggtaaaaatgatgttcctcagggcattac;

B, aagcttgcggccgctcacattccgtccttttcctttc; C, ctggaatcagtgttcttgttgc;

D, ctacaacggcagcgtattccgcctggaacagattgataaattc;

E, gtatgatgcgactattcagctgcgcctggaacagattgataaattc; F, gaatacgctgccgttgtag;

G, cagctgaatagtcgcatcatac; H, caatgccatatcgggaaacc; I, gattgagcccgaactttgaac; and

J, cacaagttcgtgcgtcaag.

The cDNA for *crh-1* was amplified using primers A and B from *cmk-1::crh-1* [5]. The amplified DNA was digested with *Age*I and *Not*I and cloned into *Age*I- and *Not*I-digested *ceh-17::dop-3fl* [13] to obtain *ceh-17::crh-1*. Next, *ceh-17::crh-1* was injected into *cat-2(e1112);crh-1(tz2)* together with a transformation marker, *lin-44::gfp* [21], which induces GFP expression in the tail, and pBlueScript (Invitrogen, Carlsbad, CA). In the aldicarb assays, the progeny of a transgenic animal were tested and those animals that carried the transgene and exhibited GFP expression were scored separately from those animals that had lost the transgene and did not show GFP expression. *cat-2(e1112);crh-1(tz2)* was injected only with *lin-44::gfp* and pBlueScript and this transgenic strain was tested as a control.

The fusion genes used for the SIA neuron-specific RNAi of the *cha-1* gene were made as described by Esposito et al [22]. Primers C and D or C and E were used to amplify the *ceh-17* promoter region from *ceh-17::dop-3*, and primers F and G were used to amplify part of the *cha-1* coding region from genomic DNA. The amplified DNAs were mixed and fusion genes were obtained using primers H and I or H and J. The fusion genes were mixed and injected into N2 and *cat-2(e1112)* mutant animals together with *lin-44::gfp* and pBlueScript. The GFP-expressing transgenic animals were used for the aldicarb assays.

### Statistical analyses

**Figure 1:**
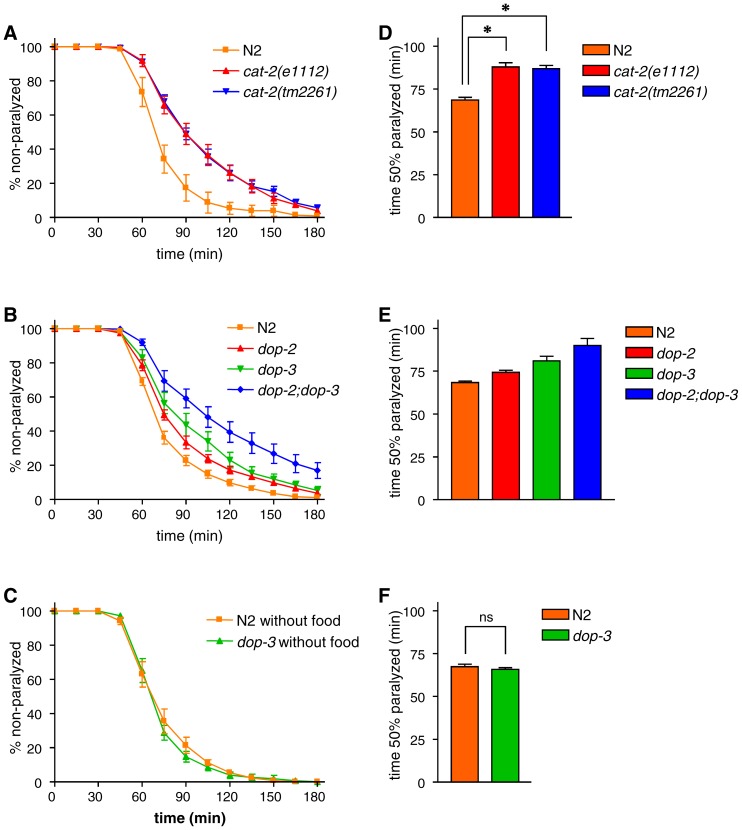
Dopaminergic mutants exhibit increased aldicarb resistance. (A-C) Animals were examined for paralysis on NGM plates containing 1 mM aldicarb. (D-F) The time required for 50% of the animals to become paralyzed was determined using Prism. (A and D) The dopamine-deficient *cat-2* mutants, *cat-2(e1112)* and *cat-2(tm2261)*, took longer to become paralyzed than wild-type N2 animals. *P<0.001 by the Tukey-Kramer multiple comparison test. (B and E) Aldicarb sensitivity of the *dop-2* and *dop-3* single mutants and *dop-2;dop-3* double mutant. Both *dop-2* and *dop-3* significantly increased aldicarb resistance (F_(1,28)_ = 9.52, p<0.01 and F_(1,28)_ = 30.23, P<0.001, respectively, by two-way ANOVA) without significant interaction (F_(1,28)_ = 0.37, P = 0.55 by two-way ANOVA). (C and F) The aldicarb resistance of N2 and *dop-3* mutant animals was measured in the absence of food. ns: P>0.05 by Student’s *t*-test. Error bars indicate the SEM.

The time required for 50% of the animals to become paralyzed (T_50_) was calculated with Prism software (GraphPad Software, San Diego, CA) by a non-linear regression analysis of the Boltzmann sigmoidal curve, as described previously for a *C. elegans* killing assay [23]. To compare the T_50_ among strains, the statistical significance was evaluated by a one-way ANOVA followed by the Tukey-Kramer multiple comparison test using Prism software, except for Figure 1E and 1F, which were evaluated by a Student’s *t*-test and a two-way ANOVA, respectively.

## Results and Discussion

### Suppression of dopamine signaling reduces acetylcholine signaling

In *C. elegans*, the relative strength of acetylcholine signaling can be measured by monitoring the paralyzing effect of the acetylcholinesterase inhibitor aldicarb [20]. Animals with a reduced level of acetylcholine signaling exhibit enhanced resistance to aldicarb, whereas animals with increased acetylcholine signaling are hypersensitive to it. To determine the effect of dopamine on acetylcholine signaling, we first analyzed the aldicarb sensitivity of *cat-2* mutants. The *cat-2* gene encodes tyrosine hydroxylase and is required for dopamine synthesis [24]. We analyzed two different alleles of *cat-2* and found that both mutants exhibited moderate resistance to aldicarb as they took significantly more time to become paralyzed than did wild-type N2 animals (Figure 1A and D). This result suggests that acetylcholine signaling was reduced in the *cat-2* mutants. The D2-like dopamine receptors DOP-2 [25] and DOP-3 [26] work downstream of dopamine in the regulation of CREB in SIA neurons [13]. We measured the aldicarb sensitivity of *dop-2* and *dop-3* single mutants as well as that of *dop-2;dop-3* double mutants (Figure 1B and E). A two-way ANOVA revealed that both the *dop-2* and *dop-3* mutations significantly increased the resistance of the animals to aldicarb (F_(1,28)_ = 9.52, p<0.01 and F_(1,28)_ = 30.23, P<0.001, respectively), whereas there was no significant interaction between the effects of *dop-2* and *dop-3* (F_(1,28)_ = 0.37, P = 0.55). These results suggest that the suppression of dopamine signaling results in reduced acetylcholine signaling.

Allen et al. reported that the *dop-3* mutant exhibits wild-type aldicarb sensitivity in the absence of food and that only in the enhanced background of *ace-1* does the *dop-3* mutation cause hypersensitivity to aldicarb [27], as opposed to resistance we observed in this study. The *ace-1* gene encodes acetylcholinesterase, which is released from muscle cells to degrade acetylcholine [28]. Therefore, *ace-1* mutants should have an increased level of acetylcholine in the neuromuscular junction. Allen et al. also showed that, for this hypersensitivity, *dop-3* works in the cholinergic motor neurons of the ventral cords [27]. The experiments in Figure 1B and E were conducted in the presence of food. Since aldicarb sensitivity is influenced by the experimental conditions [20], we also analyzed the aldicarb sensitivity of wild-type and *dop-3* animals in the absence of food and found that the *dop-3* mutant exhibited similar aldicarb sensitivity to wild-type animals in this condition (Figure 1C), which is consistent with the previous study. Our finding that *dop-3* mutants exhibit stronger aldicarb resistance than wild type animals only in the presence of food suggests that food availability influences the effect of dopamine on acetylcholine signaling. This is in line with the reports that dopamine signaling works in the presence of food to induce behavioral changes in *C. elegans* [19], [29].

### The aldicarb resistance of *cat-2* is suppressed by *tbh-1*, *ser-3*, and *crh-1*

**Figure 2:**
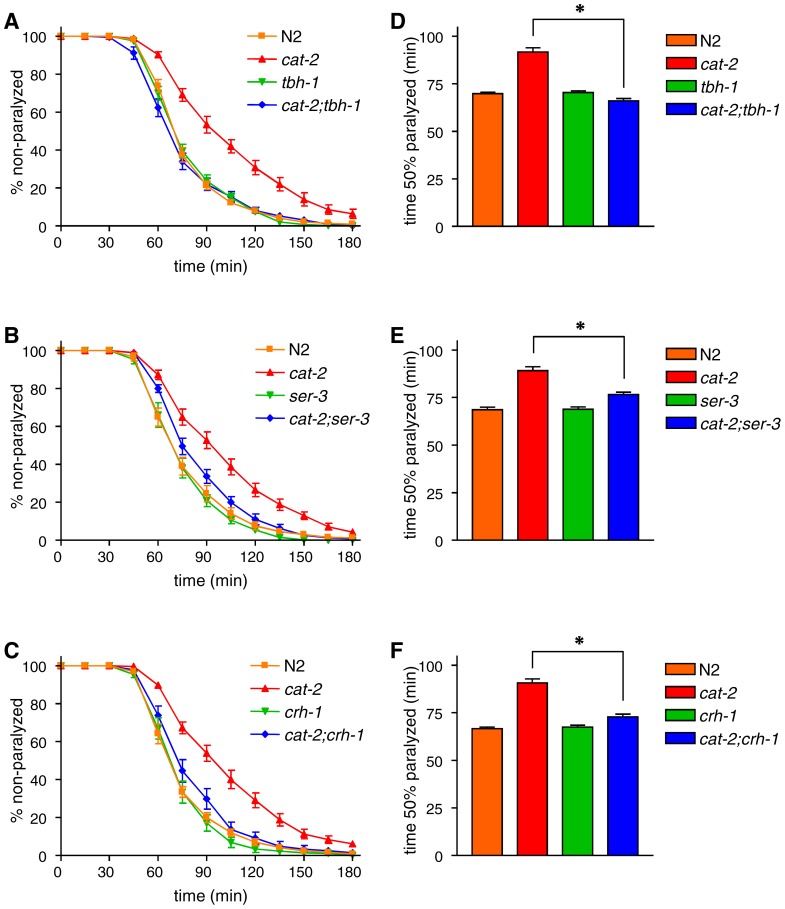
*tbh-1*, *ser-3*, and *crh-1* suppress *cat-2*. Double mutants for *cat-2* and *tbh-1* (A), *ser-3* (B), or *crh-1* (C) were examined for paralysis on NGM plates containing 1 mM aldicarb. (D-F) The time required for 50% of the animals to become paralyzed was determined using Prism. *tbh-1*, *ser-3*, and *crh-1* single mutants exhibited similar aldicarb sensitivity to wild-type N2 animals. The aldicarb resistance observed in the *cat-2* mutant was significantly reduced in the *cat-2;tbh-1*, *cat-2;ser-3*, and *cat-2,crh-1* double mutants. *P<0.001 by the Tukey-Kramer multiple comparison test. Error bars indicate the SEM.

With respect to the regulation of CREB activation in SIA neurons, octopamine signaling works downstream of dopamine [13]. We previously showed that octopamine signaling was activated in the *cat-2* mutant, and that spontaneous CREB activation in the *cat-2* mutant was suppressed by a mutation in the *tbh-1* gene, which encodes tyramine β-hydroxylase and is required for octopamine production [30]. To determine whether octopamine signaling also works downstream of dopamine in the regulation of acetylcholine signaling, we examined the effect of the *tbh-1* mutation in the regulation of aldicarb sensitivity (Figure 2A and D). The aldicarb sensitivity of the *tbh-1* mutant was not different from that of wild-type animals. The *cat-2;tbh-1* double mutant also exhibited normal aldicarb sensitivity, indicating that the aldicarb resistance observed in the *cat-2* mutant was completely suppressed by the mutation of *tbh-1*. Since the octopamine receptor SER-3 is required for octopamine-mediated CREB activation in SIA neurons [12], we next analyzed the *ser-3* mutant and found that *ser-3* also, albeit partially, suppressed the aldicarb resistance of *cat-2* (Figure 2B and E). To determine whether CREB plays a role in the regulation of acetylcholine signaling, we analyzed *crh-1* mutants. *crh-1* encodes a CREB homolog that is required for CRE-mediated gene expression [12]. *crh-1*, similar to *ser-3*, suppressed the enhanced aldicarb resistance observed in the *cat-2* mutants (Figure 2C and F), suggesting that *crh-1* also works downstream of *cat-2*. Taken together, these results suggest that the same pathway that works in the regulation of CRE-mediated gene expression in SIA neurons functions in the regulation of acetylcholine signaling.

**Figure 3:**
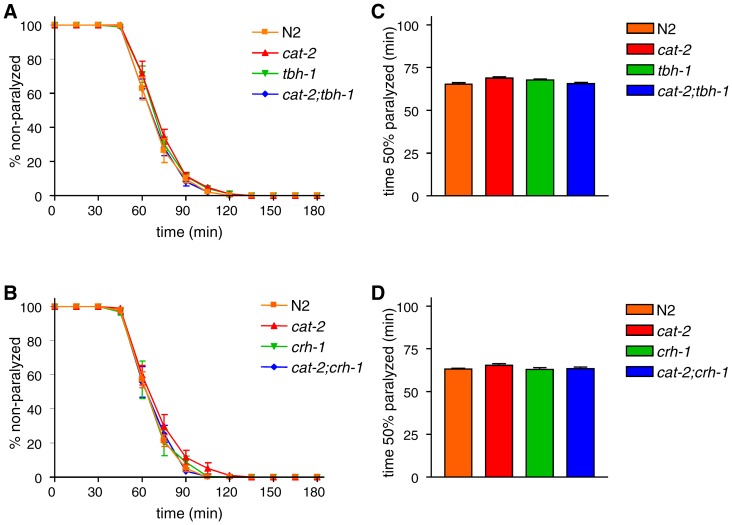
Levamisole sensitivity was unchanged in the *cat-2*, *tbh-1*, and *crh-1* mutants. (A and B) Animals were examined for paralysis on NGM plates containing 0.2 mM levamisole. (C and D) The time required for 50% of the animals to become paralyzed was determined using Prism. A one-way ANOVA revealed that *cat-2*, *tbh-1*, or *crh-1* mutation does not significantly alter levamisole sensitivity (P>0.05). Error bars indicate the SEM.

Aldicarb causes the accumulation of acetylcholine in the synaptic cleft, leading to the over-activation of cholinergic receptors on muscle and paralysis [20]. Aldicarb resistance could result from decreased acetylcholine release from neurons or from decreased acetylcholine sensitivity of the muscles. To distinguish between these possibilities, we measured the sensitivity of the mutants to levamisole, an agonist of muscle acetylcholine receptors [31]. Sensitivity to levamisole was largely unchanged in the *cat-2*, *tbh-1*, and *crh-1* mutants as well as the *cat-2;tbh-1* and *cat-2;crh-1* double mutants (Figure 3). These results suggest that the aldicarb resistance observed in the *cat-2* mutant was caused by decreased acetylcholine release from neurons rather than a change in sensitivity to acetylcholine.

### CRH-1 works in SIA neurons to regulate aldicarb sensitivity

**Figure 4:**
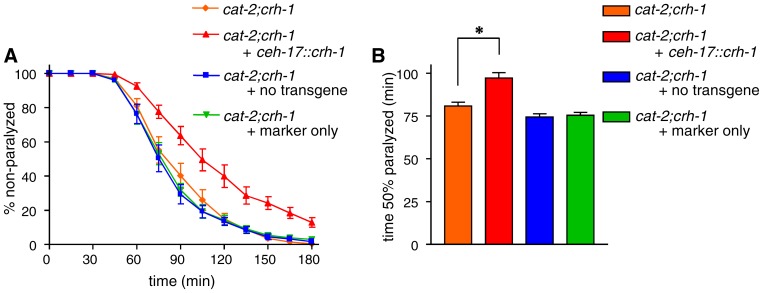
*crh-1* expression in SIA neurons rescued aldicarb resistance in the *cat-2;crh-1* double mutant. *cat-2;crh-1* mutants were transformed with *ceh-17::crh-1* and the co-injection marker *lin-44::gfp*. (A) Animals were examined for paralysis on NGM plates containing 1 mM aldicarb. (B) The time required for 50% of the animals to become paralyzed was determined using Prism. Animals carrying the transgene exhibited stronger aldicarb resistance than did the original double mutants. Those animals that lost the transgene or that were transformed only with the co-injection marker showed similar levels of aldicarb sensitivity to the *cat-2;crh-1* double mutants. *P<0.001 by the Tukey-Kramer multiple comparison test. Error bars indicate the SEM.

Using the *cre::gfp* reporter, we previously showed that SIA neurons are the only cells in which CREB activity is detectably regulated by *cat-2* [13]. To examine whether SIA neurons are indeed where CREB functions in the regulation of acetylcholine signaling by dopamine, we conducted the cell-specific rescue of *crh-1* in *cat-2;crh-1* double mutants. If *crh-1* functions in SIA neurons, the expression of *crh-1* only in these cells should increase the aldicarb resistance of *cat-2;crh-1* double mutants. For this purpose, *crh-1* was expressed under the *ceh-17* promoter (*ceh-17::crh-1*) [32], which induces gene expression only in SIA neurons and one additional neuron (the ALA neuron). The *cat-2;crh-1* double mutants carrying *ceh-17::crh-1* exhibited stronger aldicarb resistance compared to the original (*cat-2;crh-1*) double mutants, whereas control animals that had lost *ceh-17::crh-1* or that had been injected only with the co-injection marker did not (Figure 4). These results suggest that the expression of *crh-1* in SIA neurons is sufficient for the dopamine-mediated regulation of acetylcholine signaling.

**Figure 5:**
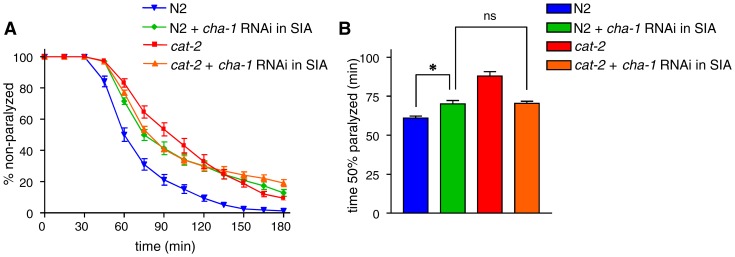
*cha-1* RNAi in wild-type and *cat-2* mutant animals. The choline acetyltransferase gene *cha-1* was specifically knocked down by RNAi in the SIA neurons of wild-type N2 animals or in the *cat-2* mutant. (A) The animals were examined for paralysis on NGM plates containing 1 mM aldicarb. (B) The time required for 50% of the animals to become paralyzed was determined using Prism. *cha-1* RNAi in SIA neurons resulted in increased aldicarb resistance in a wild-type background. The *cat-2* mutation did not significantly alter aldicarb sensitivity when *cha-1* was knocked down in SIA neurons. *P<0.001, ns: P>0.05, by the Tukey-Kramer multiple comparison test. Error bars indicate the SEM.

It remained unclear whether a change in acetylcholine release from SIA neurons contributes to the regulation of aldicarb sensitivity by dopamine. To address this, we conducted the cell-specific RNAi-mediated knockdown of the *cha-1* gene, which encodes the choline acetyltransferase and is required for acetylcholine synthesis [33], using the *ceh-17* promoter. Animals with a wild-type background that were subjected to RNAi exhibited stronger aldicarb resistance than did wild-type animals, although it was not as strong as did the *cat-2* mutant (Figure 5).Considering that the ALA neuron is not cholinergic [15], this result suggests that the removal of acetylcholine from SIA neurons alone causes aldicarb resistance. If the aldicarb resistance observed in the *cat-2* mutant was due to reduced acetylcholine release from SIA neurons, the *cat-2* mutation should have no effect in animals in which acetylcholine is removed from the SIA neurons. We therefore performed SIA neuron-specific RNAi of *cha-1* in a *cat-2* mutant background and found that the aldicarb sensitivity of this strain was not significantly different from that of animals with a wild-type background that were subjected to *cha-1* RNAi. Hence, *cha-1* RNAi did not increase the aldicarb resistance of *cat-2* but rather decreased it to the level of N2 animals subjected to *cha-1* RNAi. This result demonstrates that *cat-2* does not have any effect on aldicarb sensitivity when *cha-1* was knocked down by RNAi using the *ceh-17* promoter and suggest that the dopamine-mediated modulation of aldicarb sensitivity is dependent on acetylcholine in SIA neurons. There seems to be a tendency for RNAi strains to have a slower decline in the number of moving animals later in the assay than did *cat-2* mutants. These strains carry the transgenes as extrachromosomal arrays and it is possible that variability in the copy number of the transgene is causing a portion of RNAi animals to become more aldicarb resistance.

SIA neurons are known to be cholinergic [15] and have been shown to synapse with the head muscles in *C. elegans* [34]. However, the aldicarb assay used here measured whole-body paralysis, and the observed delay in aldicarb-mediated paralysis for the *cat-2* mutant was not limited to the head muscles; their entire body moved for a longer time after exposure to aldicarb compared with wild-type animals. In addition to connecting to the head muscles, SIA neurons possess neuronal processes that extend from the head region to the tail through the sublateral cords [34]. The function of these processes is unknown. However, neural processes in the sublateral cord contain synaptobrevin [35], which plays a role in vesicle secretion, suggesting that acetylcholine can be released from this region of the neuronal processes. SIA neurons account for only four of approximately 100 cholinergic neurons in *C. elegans*, and the muscles that control body movement are controlled mainly by ventral cord cholinergic motor neurons. Nonetheless, the results presented here suggest that reducing acetylcholine release from these four SIA neurons produces a change in aldicarb-mediated paralysis and negates the effect of *cat-2*. We used *ceh-17* promoter to express double-stranded RNA of *cha-1* with the intention to inhibit expression of *cha-1* only in SIA neurons. The cell-type specificity of *ceh-17* promoter was determined by expressing fluorescent proteins under this promoter [12], [32]. However, it remains possible that this promoter induces gene expression in other cholinergic neurons in a way that is too weak to be detected with fluorescent proteins. Such leaky expression of double-stranded RNA may be causing knockdown of *cha-1* in other cholinergic neurons. *cha-1* mutants are very uncoordinated, small, and slow growing [33]. The *cha-1* RNAi strains used in this study did not exhibit these phenotypes and therefore are unlikely to have severely reduced *cha-1* expression in many cholinergic neurons. However, a definitive way to address the involvement of SIA neurons in the amine-mediated regulation of acetylcholine release would be to laser ablate SIA neurons of N2 and *cat-2* mutants and to test them for aldicarb sensitivity as this approach does not depend on cell-specific promoters.

Exogenous application of dopamine causes reduced locomotion of *C. elegans* animals through DOP-3 [19], [36]. Furthermore, reduced dopamine clearance in the dopamine transporter mutant *dat-1* causes paralysis of animals in liquid [37]. These results suggest that dopamine signaling reduces activity of muscle, which is regulated by acetylcholine signaling. Our finding that the reduced dopamine in *cat-2* mutants causes reduced acetylcholine signaling is somewhat surprising in that dopamine is having an opposite effect on acetylcholine signaling from these previous studies. This difference may be caused by differences in when and where dopamine works. Exogenous dopamine and liquid treatment induce acute behavioral changes and exogenously applied dopamine works on the ventral cord motor neurons to control locomotion. On the other hand, it is likely that the effect of dopamine on aldicarb resistance we observed here is a slow response since it depends on a transcription factor CREB and that the SIA neurons play a role in this regulation. Our findings together with the previous reports suggest that dopamine regulates muscle activity through multiple mechanisms.

## Conclusions

Studies using several model animals have demonstrated that amine neurotransmitters regulate CREB to induce long-term changes in neuronal activity. In this study, we found that multiple dopamine signaling mutants exhibited increased aldicarb resistance, which is indicative of reduced acetylcholine signaling. Genetic experiments revealed that octopamine and CREB signaling, which is suppressed by dopamine in the presence of food, functions downstream of dopamine and that activation of this signaling pathway reduces acetylcholine release. Cell-specific rescue and knockdown experiments suggested the involvement of SIA neurons for the regulation of acetylcholine signaling by dopamine. The results of this study indicate that the regulation of CREB by amine neurotransmitter signaling modulates neurotransmitter release in *C. elegans*. Our findings will facilitate future studies of the mechanism of the CREB-mediated regulation of neurotransmitter release in the genetically tractable model organism *C. elegans*.
